# Process-Relevant Flow Characteristics of Styrene-Based Thermoplastic Elastomers and Their Representation by Rheometric Data

**DOI:** 10.3390/polym15173537

**Published:** 2023-08-25

**Authors:** Markus Kaempfe, Matthieu Fischer, Ines Kuehnert, Sven Wießner

**Affiliations:** 1 Leibniz-Institut für Polymerforschung Dresden e.V., D-01069 Dresden, Germany; kaempfe@ipfdd.de (M.K.); fischer-matthieu@ipfdd.de (M.F.); kuehnert@ipfdd.de (I.K.); 2 Institut für Werkstoffwissenschaft, Technische Universität Dresden, D-01069 Dresden, Germany

**Keywords:** thermoplastic elastomers, rheology, rheometry, testing, modeling and simulation

## Abstract

The complex multiphase morphology of thermoplastic elastomers based on styrene-block copolymers (TPSs) affects their flow behavior significantly and in a way which may not be considered by commonly used characterization and evaluation procedures. To evaluate the relevance of non-Newtonian flow phenomena for the validity of rheometric data in processing, three commercially available TPSs with comparable hardness of about 60 Shore A but with different application fields were selected and characterized using parallel plate and high-pressure capillary rheometry. The validity of the rheometric data is assessed by modeling the flow in a high-pressure capillary rheometer by a computational fluid dynamics (CFD) simulation. The results were discussed in conjunction with close-up images of samples taken after the measurement. The materials show clearly different rheological behaviors but depend on the respective shear and geometrical conditions. In particular, for the material with the lowest viscosity, doubling the capillary diameter resulted in a disproportionate increase of the pressure loss by up to one third. Only the capillary flow of this material could not be reproduced by the CFD simulation. The results indicate that conventionally determined rheometric data of TPSs are of limited use in evaluating process flows for various material grades.

## 1. Introduction

Melt processability is one of the outstanding advantages of thermoplastic elastomers (TPEs) as it enables highly efficient manufacturing technologies that provide additional design freedom for the resulting product with elastomer-like application properties. However, the complex multiphase morphology of especially blend-based TPE groups pose manufacturing challenges because it affects their flow behavior significantly. Certain process settings result in a molded part with a delaminated or ruptured surface layer or air inclusions on the product, which in particular limits its sealing ability as well as its adhesion to the material itself (weld line) and to other materials [1]. However, good adhesion is a decisive reason to choose TPEs over other materials, e.g., for multi-component injection molding of soft grips and functional elements, such as valves, seals, and closures.

One material example is the group of styrene-based thermoplastic elastomers (TPSs). They usually consist of blends of a styrene-based block copolymer (SBC) with polyolefins and additives. While the latter components serve to improve the processability, the SBC significantly determines the TPE character of the compound. It exhibits a high molecular weight olefinic midblock, which ensures rubber-like behavior and end blocks built of polystyrene, which are chemical incompatible to the midblock, and therefore affect a microphase-separated morphology. They act as glassy, physical crosslinks at service temperature. Increasing the temperature weakens the incompatibility between these two phases. However, the material remains microphase-separated at rest up to temperatures of about 400 °C [2]. The presence of ordered microdomains in SBCs of specific structure therefore inhibits the mobility of polymer chains even at temperatures typical of polymer processing, resulting in a gel-like linear viscoelastic behavior at small oscillation shear amplitudes [3] and wall slip at low-shear stresses [4]. Although their flow behavior in a hot runner may be described by considering a particular yield stress (e.g., Herschel–Bulkley model), a mixing rule [5] or combined generalized Newtonian fluid flow model, such as CARPOW [6], the determination of rheological properties needs first a breakdown of the complex flow to simplified conditions, which comes with several assumptions about the materials flow behavior, e.g., a continuum mass. Unfortunately, the available rheometric data of thermoplastic elastomers are limited and often exclude the details of the test setup and method used. Therefore, there is currently little information on whether the assumed boundary conditions are actually met in process-relevant flow processes, such as in a capillary flow, and how reliable the experimentally determined material-specific properties are for the optimization of processing conditions and for the design of hot runners, gates, sprues, and extrusion tools.

Therefore, this study aims to evaluate the relevance of non-Newtonian flow phenomena to the rheometric data of TPSs in different conventionally used experimental setups and considering different shear and geometric boundary conditions. Special attention is paid to the validity of the data derived from the high-pressure capillary rheometer, which is evaluated using computational fluid dynamics simulation.

## 2. Materials and Methods

In the present study, three commercially available grades of thermoplastic elastomers (TPEs) based on blends of styrene-block copolymers (SBCs) and polyolefins were investigated. As shown in Table 1, these materials are abbreviated as TPS-1, TPS-2, and TPS-3 in the following. The materials used are recommended for injection molding and extrusion applications and have a rather soft hardness level of about 60 Shore A. Since these are commercial grades, the composition and structure, especially the block structure of the SBCs mentioned above, are unknown. However, the investigated materials differ significantly in their applications and thus presumably in their property profiles. We therefore expect clearly different structural properties of the investigated compounds, which should allow us to cover a broad spectrum of rheological behavior typical for TPSs in a very efficient way.

The materials were rheologically characterized, considering different measurement devices and typical experimental setups used in polymer rheology. This includes the rotational rheometer ARES G2 (TA Instruments Inc. (New Castle, DE, USA)) as well as the high-pressure capillary rheometer RHEOGRAPH 2002 (GÖTTFERT Werkstoff-Prüfmaschinen GmbH (Buchen, Germany)). All measurements were performed at a temperature of 180 °C, with each material preheated for at least 4 min. Oscillatory tests were performed under nitrogen atmosphere and with smooth parallel plates of 25 mm diameter. The primary granular specimens were thereby squeezed by the movement of the upper geometry, and it was waited until the axial force leveled off to a constant value. At first, strain sweep experiments were performed at a fixed angular frequency ω of 10 rad/s to obtain a measure of the upper limit of strain amplitudes in the linear viscoelastic regime but without compromising data that can be recorded at large strains but are limited by the maximum data sampling rate and the torque range of the transducer. A strain amplitude of 0.005 has been shown to be applicable for the estimation of a complex viscosity within small amplitude oscillatory shear (SAOS) testing. The frequency sweep has been performed at the given strain amplitude, with a stepwise increase of angular frequencies ω from 0.01 to 100 rad/s. For the high-pressure capillary rheometry (HPCR) testing, different sets of capillaries were used:

- Length: *L* = 5, 10, 20, 30 mm/Diameter: *D* = 2 mm
- Length: *L* = 5, 10, 20, 30 mm/Diameter: *D* = 1 mm

Each test was prepared by first filling the feeding channel with the material and slowly moving the piston down until pressure built up and material was pushed out of the capillary. At the end of the preheating time, this procedure was repeated. The remaining preheating time was set to assure similar pressure values before and after the second piston movement, which ensures that the material is unstressed before starting the measurement. Afterwards, the piston velocity $v_{fc}$ was stepwise increased. This sets a certain volume flow in the feed channel ${\dot{V}}_{fc}$ and in the capillary ${\dot{V}}_{cap}$, which are equal and determined by the cross-sectional area $A_{fc}$ or diameter $D_{fc}$ of the feed channel, respectively, according to Equation (1a). With the use of the Hagen–Poiseuille law, these volume flows convert to apparent shear rates of ${\dot{\gamma }}_{a}=$10, 20, 40, 80, 160, 320, and 640 s^−1^ (Equation (1b)).

(1a) $${\dot{V}}_{cap}={\dot{V}}_{fc}=A_{fc}\cdot v_{fc}=\frac{\pi }{4}D_{fc}^{2}\cdot v_{fc}$$

(1b) $${\dot{\gamma }}_{a}=\frac{{32\dot{V}}_{cap}}{\pi D^{3}}$$

Each step was performed up to the point where the pressure level fulfills the stationarity criterion of a tolerance of 1% within 10 s. The corresponding pressure values were used for the further evaluation procedures by means of the viscosity estimation. In the first step, nonlinear Bagley plots were fitted to the pressure drop ∆p profiles with capillary length *L* and for the respective capillary diameters *D*, resulting in a negligible third term in the corresponding Equation (2).

(2) $$∆p=a_{0}+a_{1}\;{\left(L/D\right)}^{1}+a_{2}{\;(L/D)}^{2}+a_{3}\;{\left(L/D\right)}^{3}$$

Hence, the entrance pressure loss ${∆p}_{E}=a_{0}$ was estimated by extrapolating these quadratic functions to a zero length-to-diameter-ratio (*L*/*D*). By means of the correction proposed by Bagley [7], the pressure loss in the capillary can be estimated by the subtraction of the entrance pressure loss ${∆p}_{E}$ from the total pressure loss ∆p or by adding a correction length to the capillary length. This leads to a corrected wall shear stress $\tau_{w,B}$ following:

(3) $$\tau_{w,B}=\frac{(∆p-{∆p}_{E})D}{4L}$$

However, the volume flow and therefore the apparent shear rate are defined by the Hagen–Poiseuille law, assuming a Newtonian, parabolic velocity profile and ideal adhesion to the capillary wall. Polymer melts show pronounced shear thinning and may slip at the capillary wall. These factors were considered in the frame of this study by applying first the Mooney [8] and second the Weißenberg-Rabinowitsch [9] procedure after the Bagley correction. Corresponding formulas used for the correction of the apparent shear rate are shown in Equation (4a,b).

(4a) $${\dot{\gamma }}_{w,M}=\frac{32}{\pi {\;D}^{3}}\left(\dot{V}-\frac{\pi }{4}{\;D}^{2}{\;v}_{s}\right)$$

(4b) $${\dot{\gamma }}_{w,RW}=\frac{3}{4}\;{\dot{\gamma }}_{w,M}+\frac{1}{4}\tau_{w,B}\frac{d{\dot{\gamma }}_{w,M}}{d\tau_{w,B}}$$

where ${\;v}_{s}$ is the slip velocity. The index *w* identifies corrected values that are intended to be representative of the actual values near the capillary wall. The indices *B*, *M*, and *RW* represent the used correction method proposed by Bagley, Mooney, Weißenberg-Rabinowitsch, respectively.

In the next step, the estimated HPCR data were fitted by the Cross-WLF-model, which is defined as follows:

(5a) $$\eta =\frac{\eta_{0}}{1+{\left(\frac{\eta_{0}\;\dot{\gamma }}{\tau^{*}}\right)}^{1-n}}$$

(5b) $$\eta_{0}=D_{1}\;exp\left[-\frac{A_{1}\;\left(T-T_{0}\right)}{A_{2}+\left(T-T_{0}\right)}\right]$$

(5c) $$T_{0}=D_{2}+D_{3}\;p\;\;\;\mathrm{and}\;\;\;A_{2}=A_{3}+D_{3}\;p$$

where

- η and $\eta_{0}$ are the shear and zero-shear viscosity,
- $\tau^{*}$ and n are adjustable parameters, where $\tau^{*}$ is the critical shear stress at the transition to shear thinning and n its power law index in the high shear rate region,
- T and $T_{0}$ are the temperature and reference temperature,
- and $D_{1},\;D_{2},\;D_{3},\;A_{1},\;A_{3}$ are the fitted coefficients, respectively.

The aim of the work was a fundamental investigation. In order to minimize the influence of dissipative heating and pressure on viscosity, the pressure levels in the HPCR experiments were kept as low as possible. For this reason, apparent shear rates up to 640 s^−1^ were covered and a capillary geometry with sufficient but as small as possible *L*/*D* was preferred for modeling. For a capillary with a length of 20 mm and a diameter of 2 mm, it was found that for the used geometry, only small temperature increases due to dissipative heating could be detected in the selected measurement range (∆*T* < 0.3 K) and the pressures could be minimized (∆*p* < 10 MPa). Considering only minor viscosity changes in this setup, the parameters stated in Equation (5c) can be reduced to an isobaric ${(D}_{3}=0)$ and isothermal case.

According to this approach, the required WLF parameters are obtained from a single database entry (TPS-2) for all studied materials. They are listed in Table 2. The rheological model parameters ($\eta_{0},\tau^{*},n$) were adjusted accordingly to the measured rheometric data.

**Figure 1:**
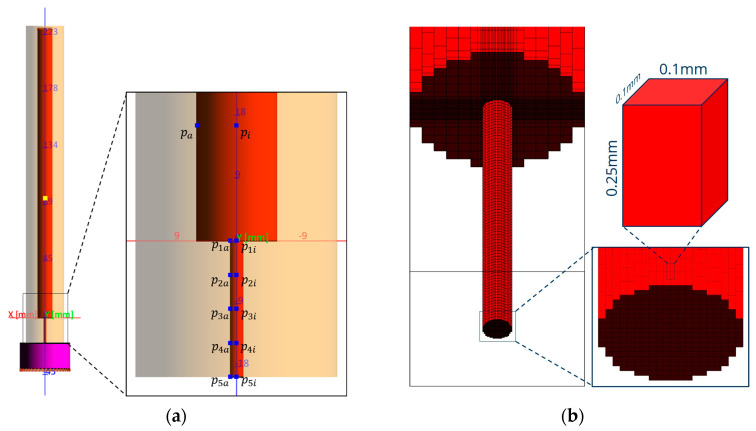
(**a**) CAD model of the HPCR setup and (**b**) the mesh geometry used in this work.

For the computational fluid dynamics (CFD) simulation, the software package SIGMASOFT^®^ was used. A CAD model with the appropriate dimensions of the used HPCR setup was created and meshed using a finer mesh in the capillary region. Important positions in the model and the corresponding mesh are shown in Figure 1.

The inflow was kept at a constant volume flow and placed at the inlet position of the feeding channel of the rheometer. In the CAD model, a large filling volume was defined after the exit of the capillary (Figure 1a, colored purple) since a finite filling volume is required for the 3D injection molding simulation program. The outflow was kept at the bottom of this defined volume. A sequence of volume flows was used, equivalent to the sequence of piston velocities in one measurement of TPS-2. Their calculation is based on Equation (1a). The wall slip was not explicitly included in the simulation calculation, i.e., only the default settings of the software were used, which stores a certain adhesion factor for the calculation.

## 3. Results

### 3.1. Rheological Behavior at Small and Large Shear Strains

**Figure 2:**
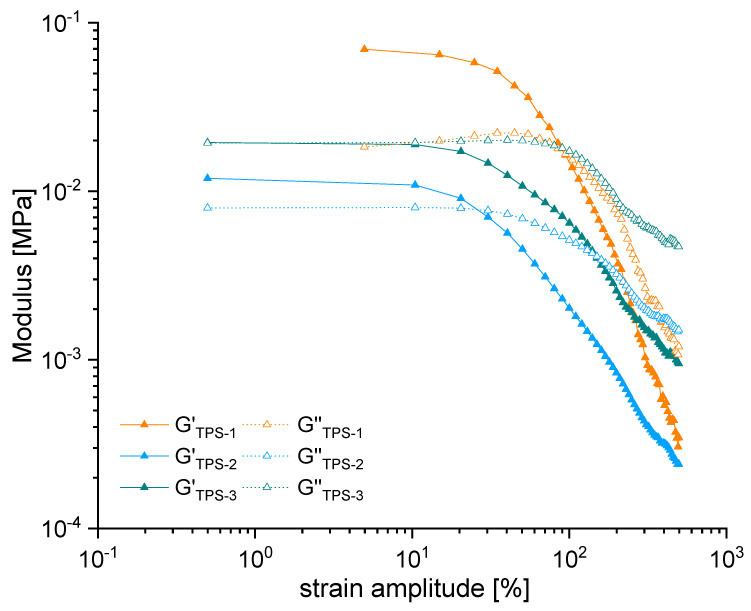
Oscillatory shear amplitude sweep results (*G*′: storage modulus, *G*″: loss modulus). Lines are drawn to guide the eye.

The results of the oscillatory shear amplitude sweeps are shown in Figure 2. Comparing the Storage Modulus *G*′ with the Loss Modulus *G*″ at low-strain amplitudes, differences among the three studied TPS are apparent. The materials TPS-1 and TPS-2 show a *G*′ noticeably larger than the *G*″ at low-strain amplitudes, indicating a predominantly elastic behavior. The storage modulus *G*′ decreases rapidly at medium-strain amplitudes, accompanied with a less pronounced *G*″ decrease. Above a critical strain, the material starts to flow, characterized by a predominantly irreversible deformation (*G*′ *< G*″). In TPS-1, this transition between different deformation regimes is accompanied by a local *G*″ maximum, indicating a dissipative mechanism. Such behavior is not evident in the TPS-2 material. However, if the strain amplitude is further increased, the viscoelastic behavior is dominated by the viscous response (*G*′ *< G*″), whose contribution increases with increasing strain amplitude. In contrast to TPS-1 and TPS-2, the TPS-3 material exhibits this behavior over the entire strain amplitude range studied.

The data presented in Figure 2 indicate that the materials TPS-1 and TPS-2 exhibit rest structures, which inhibit flow at small deformations considerably. In this work, their contribution is considered by a yield behavior at a certain shear stress in the oscillation test but is not included in the modeling and simulation. With this approach, experimental and numerically generated data for pressure-driven flows can subsequently be evaluated with respect to the expression of yield stress-driven phenomena, e.g., plug flow, without compromising the validity of the approach. Two approaches to estimate the yield stress from oscillatory shear data are used, inspired by the review of Castro et al. [10]. The procedures and estimated values are presented in Table 3.

TPS-1 shows good agreement of yield stress values resulting from both approaches. TPS-2 shows a much higher yield stress estimated by the procedure of Shih et al. [13]. The latter yield stress value is used for the further evaluations of the numerical results since it is of interest to represent the stress at which the material begins to flow in a practical sense rather than the stress at which elastic network structures start to break. In order to make this value as applicable as possible for steady-state flow conditions (as in the HPCR method), we decided to use effective shear stress values instead of amplitude shear stress values for further evaluations.

**Figure 3:**
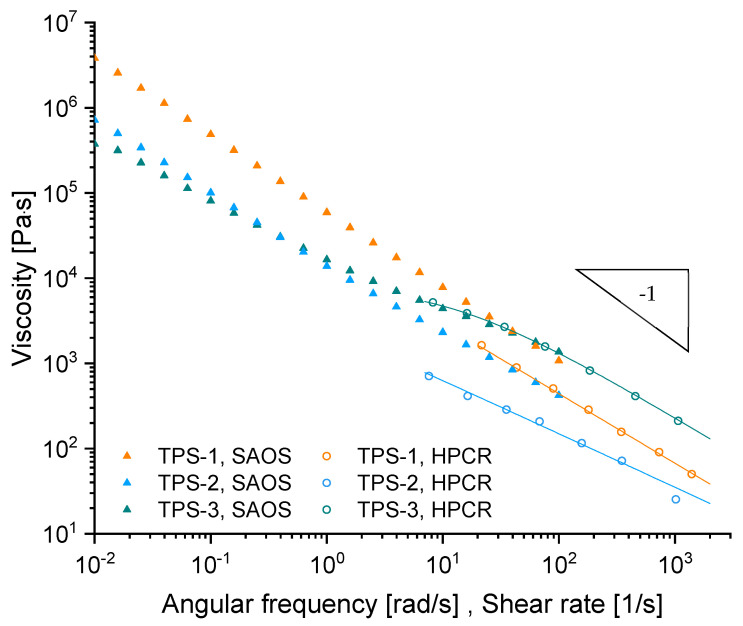
SAOS and HPCR data (T = 180 °C, 20/2 capillary). Lines—Cross-WLF fit corresponding to the parameters listed below.

As shown in Figure 3, the oscillatory data of TPS-3 are in good agreement with the capillary data, effecting an applicable Cox–Merz relation [14]. In contrast, TPS-1 and TPS-2 show a noticeable shift between viscosity values of the SAOS and HPCR data. This is usually associated in the literature either with wall slip [15] and/or a pronounced sensitivity of the rheological properties to the applied shear strain [16] as is common for highly filled polymers [17].

The viscosity curves (HPCR) of the studied materials are fitted by the Cross-WLF equation (Equation (5a,b)). Resulting fitting parameters are summarized in Table 4.

The viscosity curves obtained by HPCR measurements of the materials TPS-1 and TPS-2 (Figure 3) do not show a Newtonian plateau in the studied shear rate range. Hence, an accurate fit could only be achieved by a comparably large $D_{1}$ parameter corresponding to a zero-shear viscosity at reference temperature at least ten times higher than that measured with the HPCR method. The model parameters $D_{1}$ and $\tau^{*}$ of these materials do not correspond to the flow properties in terms of a definite physical interpretation but are sufficient to be used in the numerical approach presented—limited to temperatures close to the reference temperature and to shear rates within the selected range.

The studied materials, especially TPS-1 and TPS-2, show pronounced shear thinning within the HPCR testing, indicated by very low values of the power law index n. However, the HPCR data of the TPS systems studied do not indicate a purely plug-like velocity profile (n→0) within the capillary, as obtained for several SBCs [4]. The HPCR data of the studied TPS systems do not support a pure plug-shaped velocity profile within the capillary but seem rather close to it depending on the type of material in question.

### 3.2. Effect of Capillary Diameter (D) on Rheometric Data

**Figure 4:**
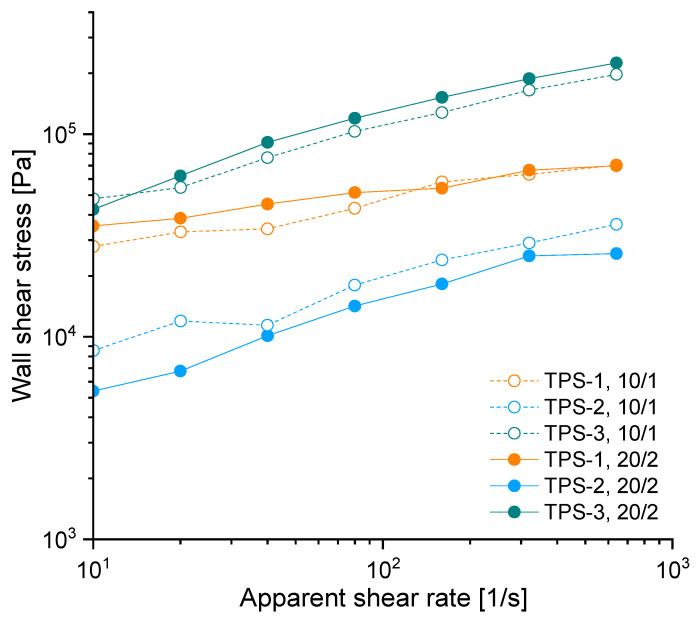
Bagley-corrected wall shear stress for capillaries with constant *L*/*D* ratio and different diameters. Lines are drawn to guide the eye.

A plot, which is usually used to evaluate the contribution of wall slip and plug flow on capillary data, is shown in Figure 4. According to the method proposed by Mooney [8], a material shows wall slip, if in this plot the wall shear stress in capillaries with smaller diameter is smaller than the one with a capillary of bigger diameter but equal *L/D* ratio, assuming a constant pressure and/or shear stress level. This is attributed to the higher contribution of wall slip to the estimated shear stress and rate because the wall-to-volume-proportion increases with increasing capillary diameter. Contrary, the wall shear stresses of plug-flowing materials are commonly associated with the opposite trend [16].

Accordingly, TPS-1 (at small shear rates) and TPS-3 would show wall-slip and TPS-2 a plug flow behavior in the capillary. However, the data shown in Figure 4 are not sufficient to interpret the contribution of plug flow or wall slip to the estimated rheological properties. All geometry-dependent, overlapping rheological effects contribute to the total pressure loss and may therefore affect estimated shear stresses and/or rates.

**Figure 5:**
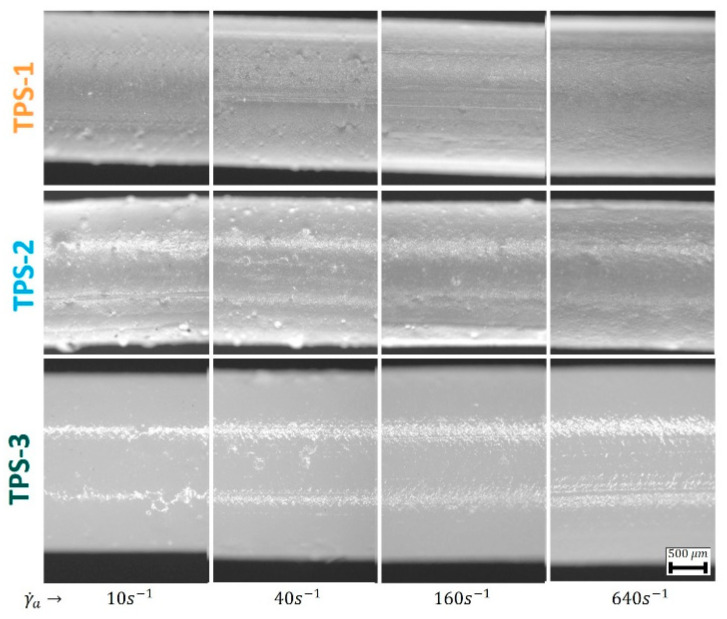
Close up images of strands extruded at different apparent shear rate values with the (20/2) capillary, cooled down to room temperature in air.

Materials with good adhesion to the capillary wall usually show rough surfaces after extrusion through a die [18]. In contrast, wall slip should result in almost flat, glossy surfaces [18]. Figure 5 shows images of the strands extruded through the 20/2 capillary at different piston velocities (corresponding to the respective apparent shear rates).

The strands of TPS-3 show very glossy surfaces for all conditions, especially at low shear rates. The gloss decreases slightly with increasing shear rate. Thus, the surface appearance of the extruded strands is indicative for the presence of wall slip of TPS-3, as shown in Figure 4. In contrast, the appearance of TPS-2 is more dependent on the applied shear rate. At small apparent shear rates, TPS-2 shows a glossy surface. At the highest apparent shear rate, TPS-2 strands show matte, rough surfaces. High wall shear stresses initiate the disruption of permanent networks of SBCs [3], which causes an increasing adhesion to the wall [4]. This seems applicable in a way to the studied TPS-2 blend, which has shown an elastically dominated viscoelastic response at small shear amplitudes (Figure 2). The strands of TPS-1 show matte, rough surfaces for all conditions, which does not indicate wall slip, contradictory to the trends obtained from Figure 4 at small shear rates.

### 3.3. Effect of Capillary Length (L) on Rheometric Data

**Figure 6:**
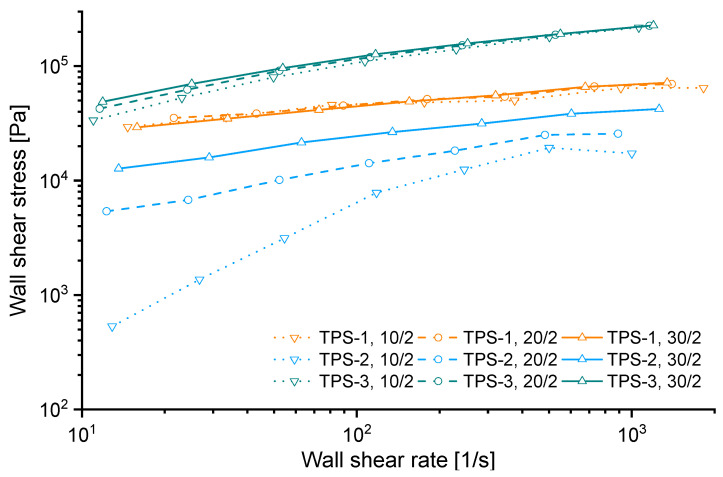
Bagley and Weißenberg-Rabinowitsch-corrected data of capillaries with different lengths and a diameter of 2 mm. Lines are drawn to guide the eye.

Figure 6 shows the flow curves for capillaries with a constant diameter (*D* = 2 mm) and different lengths. For TPS-1, the different capillary lengths lead to a wider spread of data. TPS-3 shows a slight influence of capillary length at low shear rates. However, the effect of capillary length on the data appears to be negligible for TPS-1 and TPS-3 materials overall, suggesting that their rheological behavior in the given experimental setup can be described quite well by steady-state shear material properties (and by a small additional contribution from wall slip, as shown in Figure 4).

For TPS-2, the estimated wall shear stress increases noticeably with increasing capillary length. This behavior is more pronounced the lower the estimated wall shear rate or dwell time in the capillary. With the presented data so far, it is not sufficient to attribute the rheological behavior of TPS-2 to steady-state shear properties alone.

The origins of the very different rheological behaviors of TPS-2 and TPS-1 need to be further investigated. However, for the TPS-2 material, which has both a yield stress and the lowest viscosity level of the studied compounds overall, it could be argued that the shear stresses compete more strongly with the yield stress than it seems to be the case for TPS-1, hence developing a more plug-like velocity profile within the capillary. However, since the boundary conditions under which the data were obtained appear to be unsteady for TPS-2, it is not possible to make a definite statement about the flow behavior and especially about wall shear stresses in the capillary based solely on the pressure data from the capillary rheometer recorded at the pressure transducer position in front of the entrance region of the capillary.

**Figure 7:**
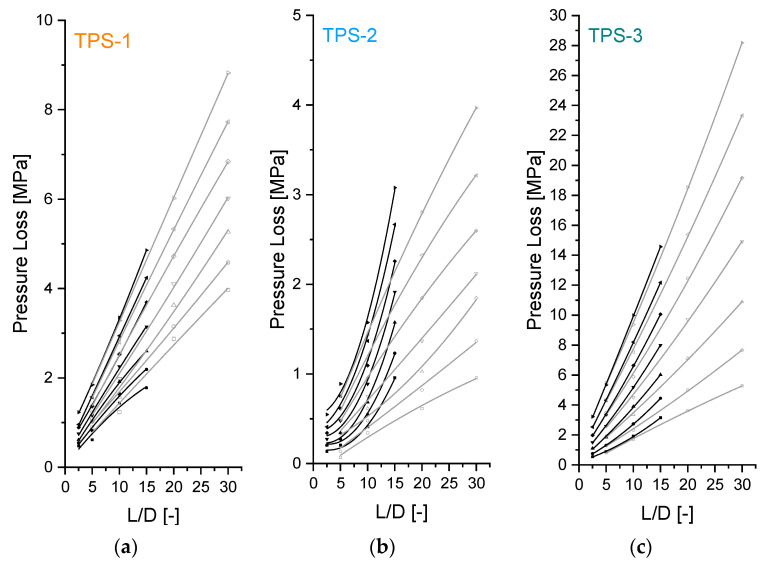
Nonlinear Bagley plots estimated from capillary sets with different lengths each of (**a**) TPS-1, (**b**) TPS-2, and (**c**) TPS-3 (grey: *D* = 1 mm, black: *D* = 2 mm).

However, the rheometric data can also be evaluated with respect to the occurring rheological effects by means of a Bagley plot, paying attention to comparisons of pressure drops of certain capillary dimensions at constant shear stress levels or length–diameter ratios (*L*/*D*). Bagley plots of the studied TPS are shown in Figure 7. It is worth pointing out that they summarize raw pressure data, including entrance pressure losses, which can differ in the two corresponding capillary diameters considerably.

The Bagley plots of the capillaries with *D* = 1 mm look almost linear, which indicates a linear pressure loss along the die and a steady-state flow pattern. Using this capillary setup, steady-state shear viscosity data can be estimated for the studied TPS in a very reliable way. However, the materials show very different patterns of the total pressure loss with increasing length-to-diameter-ratio (*L*/*D*) at larger capillary diameter. The Bagley plots of TPS-3 become more convex at the capillary with larger diameter (*D* = 2 mm), which affects higher pressure losses, especially at large *L*/*D*. This effect does not have a clear trend with increasing volume flow rate, indicating overlapping rheological effects. For example, wall slip in the capillary with smaller diameter can lead to smaller pressure losses and thus a slightly concave pressure profile (see Figure 7c: Bagley plot at *D* = 1 mm at the lowest volume flow rate). However, these pressure losses are compensated for at high volume flow rates, e.g., by the higher overall pressure level. The capillary geometry seems not decisive to pressure loss gradients by means of a shear stress estimation of the TPS-3. This may also apply to the material TPS-1 in some way. At moderate volume flow rates, the material shows comparable curves of the Bagley plots with increasing *L/D* for both capillary diameters. They seem almost linear. However, differences at low volume flow rates occur. The pressure data spread noticeably, which indicates a low reproducibility of the HPCR measurements and a high sensitivity of the material to the prehistory of deformation. Indeed, S-shaped Bagley plots were estimated previously for materials exhibiting permanent networks with a three-dimensional propagation and found that their shape is affected by changing the preshear conditions in the feeding channel by keeping another piston geometry [19]. The material TPS-1 shows overall concave Bagley plots (*D* = 2 mm) at low volume flow rates. As previously discussed in context with Figure 4, this may indicate wall slip—a decrease in pressure losses the higher the *L/D* and the smaller the capillary diameter.

The material TPS-2 shows a pattern of Bagley plots that is very different from the other studied materials. Despite that the Bagley plots estimated from capillaries with 1 mm diameter are almost linear, convex Bagley plots are achieved using capillaries with 2 mm diameter. This is also reflected by the previously discussed wall shear stress data shown in Figure 6. It suggests a pronounced sensitivity of the rheological properties to the boundary conditions of the flow. Since the pressure transducer is positioned in front of the entrance region of the capillary, the measured total pressure loss provides integrative information that includes not only all rheological effects but also the flow processes within different regions of the HPCR, i.e., from the feeding channel to the exit of the capillary. Although they cannot be determined separately, they may arguably affect each other in ways not accounted for by commonly used characterization and evaluation procedures. For example, in addition to the well-known pressure effects on viscosity, nonlinear pressure build-ups with increasing *L*/*D* can also be associated with the following:

- Increasing normal stresses along the flow path length, originated from the relaxation of the recoverable strains of the entrance [20],
- A decreasingly plug-like velocity profile with increasing flow path length in the capillary [21].

### 3.4. Experimental and Numerically Predicted Pressure Drops

**Figure 8:**
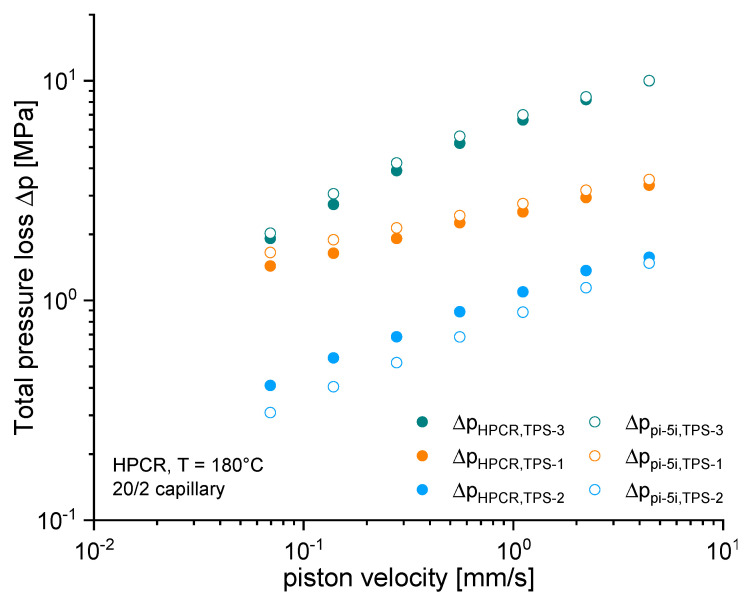
Experimental and numerical results of the total pressure loss (from pressure sensor position pi to outflow position p5i).

Figure 8 shows the total pressure loss estimated from the HPCR procedure (filled symbols) and the one predicted by the numerical simulation (open symbols) for the experimentally targeted (apparent) volume flow rates.

For the materials TPS-1 and TPS-3, experimentally estimated pressures are slightly smaller than the numerically predicted ones, which, however, is rather decisive to pressures at low apparent volume flow rates. As discussed in the previous section, this may reflect the tendency of these TPSs to slip at the capillary wall. However, the TPS-1 and TPS-3 materials show overall very good agreement between the pressures determined by the HPCR method and those predicted by the viscous Cross-WLF model and the given boundary conditions. Clear differences in Figure 8 can only be seen for the material TPS-2. Here, the viscous model clearly underestimates the pressure, which is particularly pronounced at low apparent shear rates. As discussed in conjunction with the flow curves (Figure 6) and Bagley plots (Figure 7b), an additional pressure demand builds up, which is rather decisive to the overall pressure loss at low apparent shear rates. Obviously, Figure 8 suggests that this additional pressure demand cannot be described by the Newtonian assumptions used for the evaluation of HPCR data.

### 3.5. Yield Behavior and Capillary Flow

**Figure 9:**
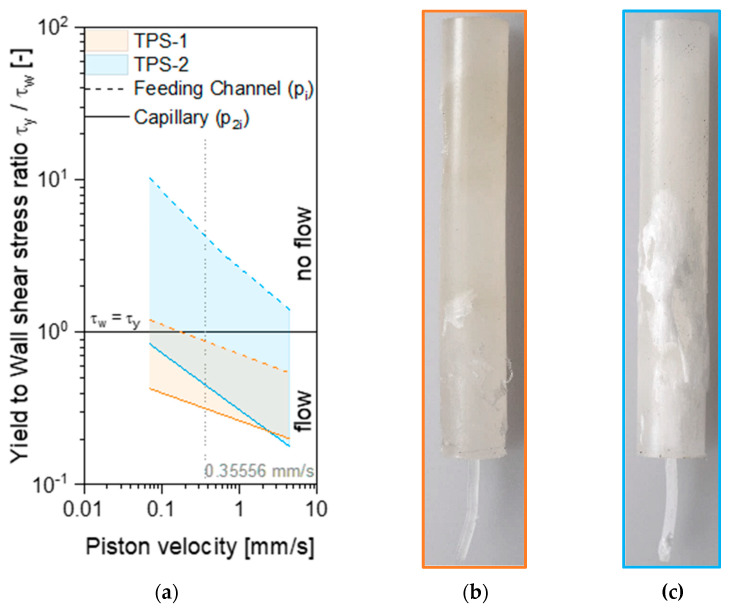
(**a**) Extent of plug flow (using a Bingham number) in different sections of the HPCR and (**b**,**c**) samples after completion of a single measurement at a piston velocity of 0.35556 mm/s *(*${\dot{\gamma }}_{a}=80\;s^{-1}$*)* at a position of 70 mm and cooling in the rheometer (extraction at 50 °C).

The numerical predicted shear stresses are now rescaled by the estimated yield stress for both materials by defining a ratio between yield and obtained shear stresses (Bingham number), which is applicable to evaluate the extent of plug or shear flow. Figure 9a depicts the determined stress ratio over piston velocities for different sections within the HPCR device. While for TPS-1, the shear stresses in the feeding channel should be high enough to shear the material to some extent, the simulated shear stress level of TPS-2 in the feeding channel is significantly lower than the yield stress, which indicates a dominating plug-flow behavior. In contrast, the shear stresses of TPS-2 in the capillary are higher than yield stress, causing shear flow of the material. These results stay in good agreement with previous shown experimental data, which suggest a plug and/or unsteady flow of TPS-2. They are also confirmed by the images of the samples produced after extrusion at a piston velocity of 0.35556 mm/s. While the TPS-1 material shows no particular surface peculiarities (apart from some ruptured surfaces), the surface of the TPS-2 sample shows an opaque layer that was torn off the surface during sample extraction. The formation of a polyolefinic and oily skin layer has already been observed for TPS, especially for extrudates from HPCR as well as for injection-molded plates [22]. Since the formation of a skin layer is a time-dependent phenomenon, the extracted sample of TPS-2 shows a skin layer only at sufficiently long residence times in the feeding channel, as shown in Figure 9c. In addition, the surfaces of the extruded strands (Figure 9b,c) readily support the formation of a polyolefin and oil-containing skin layer as they exhibit a very shiny surface with bubbles, especially at low apparent shear rates corresponding to low volumetric flow rates and consequently much higher dwell times in the capillary. However, the Bagley plots already shown in Figure 7b argue against the formation of a low-viscosity skin layer since the curve slope (and hence the determined wall shear stress) increases with increasing *L/D* and hence residence time in the capillary. Furthermore, the apparent plug flow of TPS-2 depicted by the flow curves for different capillary diameters (Figure 4) is hardly supported by the extent of plug flow calculated in the capillary by using a Bingham number. It seems not sufficient to attribute the obtained rheological behavior either to plug flow or time-dependent flow phenomena alone.

## 4. Discussion

In this study, commercially available TPS grades were characterized using conventional rheometric measuring equipment and procedures. Rheological effects were considered by comparing rheometric data of different capillary geometries as well as images of strands extruded by the HPCR. Wall slip effects appear in the rheometric data addressed in this work. However, they seem to have little effect on the determined rheological properties and the numerically predicted total pressure drops of the TPS-1 and TPS-3 materials at the HPCR. The TPS-3 material exhibited the highest viscosity at moderate shear rates of the materials studied and showed an almost viscous response to small amplitude oscillatory shear. The application of larger strain rates generally had little effect on the flow behavior of the material, so the Cox-Merz relationship was applicable. In contrast, the response of TPS-1 and TPS-2 materials to small shear amplitudes is largely governed by their elasticity. A significantly lower viscosity level was determined for these materials when the HPCR procedure was used. The application of higher strain amplitudes or rates transforms the deformation pattern of these materials from a viscoelastic gel to a viscoelastic liquid. This transition is particularly pronounced in the TPS-1 material. However, the rheological behavior of this material in the HPCR system was easily accounted for (and numerically predicted) by steady-state shear flow properties, namely a very high zero shear viscosity and a pronounced shear thinning.

In contrast, the transition behavior appears to affect the flow of TPS-2 within the HPCR markedly. The pronounced shear stress gradient between the feeding channel and the capillary indicates that the transition from a viscoelastic gel to a viscoelastic liquid takes place near the capillary entrance, imposing additional pressure requirements that cannot be reproduced by the HPCR correction procedure and the resulting steady-state shear properties alone. However, the reason for the observed behavior remains unclear, especially because it is not possible to distinguish between locally affected pressures outside the pressure drop right before the capillary entrance region and further evaluation procedures assuming a length (and therefore pressure- and dwell time) independent entrance pressure drop. Especially for materials with such different deformation behavior at low and high shear stresses, one should not conclude that these independently affect the pressure-driven flow in several sections of the HPCR. This needs to be investigated in more detail, e.g., by further CFD simulations covering larger capillary dimensions as well.

## 5. Conclusions

The results indicate that conventionally derived rheometric data of TPS materials may be of limited use for the simulation of processing flows, depending on the dimensions of, e.g., runners, molds, gates, and the respective material grade. Furthermore, rheometric data cannot be expected to reflect very well the rheological properties of a pre-plasticized TPS compound during extrusion or injection molding as evidenced by the sensitivity of flow behavior to shear history in the research literature [3,23]. It is therefore even more important to come as close as possible to the processing conditions with the boundary conditions of the rheological characterization. In particular, as stated in the VDI Guideline 2020 [24], the HPCR is the best-suited method for the rheological characterization of TPS on a laboratory scale. However, further investigations are required at this point, which should also focus on rheological characterization according to a process-related history.

## Figures and Tables

**Table 1:** Nomenclature and application field of used TPS materials.

Abbreviation	Application Field	Property Profile
TPS-1	Seals, valves, closures, and flexible connections for consumer daily life	High elasticity, excellent adhesion to PP
TPS-2	Soft-touch components for consumer daily life	Good adhesion to PP
TPS-3	Seals and functional parts with contact to drinking water	Good adhesion to PP/PE

**Table 2:** WLF parameters used for rheological modeling of all materials studied in this work.

WLF-Parameter	TPS-2
$A_{1}=$	3.8698
$T_{0}=D_{2}=$	483.15 K
$A_{2}=A_{3}=$	187.7341 K

**Table 3:** Procedures and results for the yield stress determination from amplitude sweep oscillatory data (Figure 2).

**According to**	Yang, Youssry et al. [11,12]	Shih et al. [13]
	Maximum in the plot of	Crossover point
Estimation procedure	$\tau_{0}=\gamma_{0}G_{0}^{\prime }$ versus $\gamma_{0}$	$\tau_{0}{|}_{G^{{}^{\prime }}=G^{\prime \prime }}=\frac{G^{\prime }\cdot \gamma_{0}}{cos\;\delta }{|}_{G^{\prime }=G^{\prime \prime }}$
	Effective yield stress τy=τ02 [kPa]	
TPS-1	13.91	13.07
TPS-2	1.61	5.52
TPS-3	-	-

**Table 4:** Fitting parameters corresponding to the Cross-WLF fit.

Cross-Parameter	TPS-1	TPS-2	TPS-3
n=	0.184	0.37	0.18
$\tau^{*}=$	2503.1724	650	1,750,000,000
$D_{1}=$	70,000,000	15,000	4200

## Data Availability

All data presented are only available after request from the corresponding author assuming a formal approval of respective company partners.
